# Germ Cell Nuclear Factor Regulates Gametogenesis in Developing Gonads

**DOI:** 10.1371/journal.pone.0103985

**Published:** 2014-08-20

**Authors:** Davood Sabour, Xueping Xu, Arthur C. K. Chung, Damien Le Menuet, Kinarm Ko, Natalia Tapia, Marcos J. Araúzo-Bravo, Luca Gentile, Boris Greber, Karin Hübner, Vittorio Sebastiano, Guangming Wu, Hans R. Schöler, Austin J. Cooney

**Affiliations:** 1 Department of Cell and Developmental Biology, Max Planck Institute for Molecular Biomedicine, Münster, Germany; 2 Department of Molecular and Cellular Biology, Baylor College of Medicine, Houston, Texas, United States of America; 3 Centre for Inflammatory Diseases and Molecular Therapies, The University of Hong Kong, Pokfulam, Hong Kong; 4 INSERM, U693, Faculté de Médecine Paris-Sud, Paris, France; 5 Center for Stem Cell Research, Institute of Biomedical Sciences and Technology, Konkuk University, Seoul, Republic of Korea; 6 Department of Neuroscience, School of Medicine, Institute of Biomedical Sciences and Technology, Konkuk University, Seoul, Republic of Korea; 7 Group of Computational Biology and Systems Biomedicine, Biodonostia Health Research Institute, San Sebastián, Spain; 8 Institute for Stem Cell Biology and Regenerative Medicine, Stanford University School of Medicine, Palo Alto, California, United States of America; 9 Medical Faculty, University of Münster, Münster, Germany; National University of Singapore, Singapore

## Abstract

Expression of germ cell nuclear factor (GCNF; Nr6a1), an orphan member of the nuclear receptor gene family of transcription factors, during gastrulation and neurulation is critical for normal embryogenesis in mice. Gcnf represses the expression of the POU-domain transcription factor Oct4 (Pou5f1) during mouse post-implantation development. Although *Gcnf* expression is not critical for the embryonic segregation of the germ cell lineage, we found that sexually dimorphic expression of *Gcnf* in germ cells correlates with the expression of pluripotency-associated genes, such as *Oct4*, *Sox2*, and *Nanog*, as well as the early meiotic marker gene *Stra8*. To elucidate the role of Gcnf during mouse germ cell differentiation, we generated an *ex vivo Gcnf*-knockdown model in combination with a regulated CreLox mutation of *Gcnf*. Lack of Gcnf impairs normal spermatogenesis and oogenesis *in vivo*, as well as the derivation of germ cells from embryonic stem cells (ESCs) *in vitro*. Inactivation of the *Gcnf* gene *in vivo* leads to loss of repression of *Oct4* expression in both male and female gonads.

## Introduction

Germ Cell Nuclear Factor (GCNF), also known as nuclear receptor subfamily 6, group A, member 1 (Nr6a1), is an orphan member of the nuclear receptor (NR) gene family of ligand-activated transcription factors [1]. Gcnf exhibits distinctive DNA-binding properties. Recombinant Gcnf binds as a homodimer to a response element, a direct repeat with zero base-pair spacing, i.e., a DR0, to repress the expression of genes both *in vivo* and *in vitro* [1]–[5]. *In vivo*, Gcnf appears to be part of a large complex termed transiently retinoid induced factor (TRIF) that binds to DR0 DNA elements in P19 embryonal carcinoma cells (ECCs) or embryonic stem cells (ESCs) [2], [6], [7]. In the mouse, *Gcnf* is expressed in the developing nervous system, placenta [8], [9], embryonic gonads, and adult ovaries and testes [1], [10], [11]. It is also expressed in round spermatids in mouse and in spermatocytes undergoing meiotic prophase in human [1], [10]–[12]. Gcnf has been found to regulate the transcription of the protamine genes *Prm 1* and *Prm 2* in mouse testis, antagonizing the effects of CREM tau by binding to DR0 elements in the promoters of these two genes, consistent with a role in regulating adult male fertility [4], [13], [14]. *Gcnf* is also expressed in the oocytes of vertebrates such as zebra fish, Xenopus, and mouse [1], [10], [15], [16]. Mutation of *Gcnf* specifically in oocytes using Cre/lox technology and ZP3Cre reduces female fertility [17]. Expression of *Gcnf* in gastrula- to neurula-stage embryos is critical for normal embryogenesis, as loss of this gene leads to embryonic lethality on embryonic day (E) 10.5 due to multiple defects, including placental and cardiovascular defects, posterior truncation, and disruption of normal somatogenesis and formation of the neural tube [8], [18], [19]. Gcnf acts as a repressor of the POU-domain transcription factor Oct4, a protein essential for the maintenance of the mammalian germline, and other pluripotency-associated genes during mouse post-implantation development. *Gcnf*-mutant embryos exhibit failure to repress *Oct4* expression after gastrulation [2], [6]. Gcnf is essential in the repression and silencing of *Oct4* expression during the differentiation of ESCs [6], [7], [20]. Gcnf-dependent repression of *Oct4* expression is mediated by Gcnf binding to an evolutionarily conserved DR0 element, located in the *Oct4* proximal promoter [2]. As *Oct4* is required for the survival of primordial germ cells (PGCs) [21], the question arises as to whether Gcnf plays a role in the segregation or maintenance of the PGC lineage. To address this question, we developed new mouse models and *in vitro* cell models to study the role of Gcnf in PGCs.

We therefore conducted mechanistic studies to determine the requirement of Gcnf for germ cell development during mammalian development, particularly during meiosis, which represents a critical checkpoint in the formation of normal gametes. Progression of *in vitro*–derived germ cells through meiosis is still a rare phenomenon and poses a huge challenge for reproductive medicine. Studies on gene regulation in germ cells should enhance our understanding of the mechanisms underlying meiotic processes *in vivo*, subsequently enabling us to enhance the progression of germ cells through meiosis *in vitro*. As Gcnf plays an essential role in gene regulation during early mammalian development, this study focused on elucidating the function of Gcnf during the development of murine germ cells.

## Results

### Gcnf is not required for the segregation of germ cell lineage

**Figure 1:**
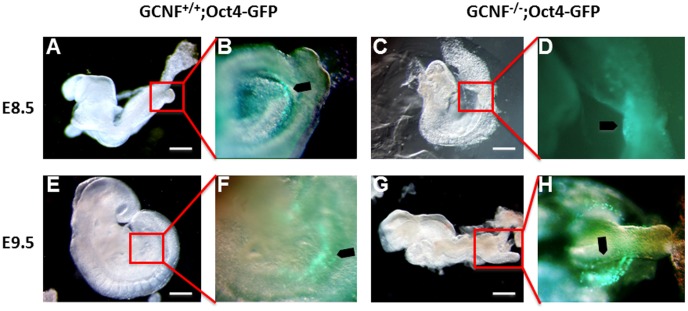
Detection of PGCs expressing green fluorescence from Oct4-GFP(ΔPE) in wt and *Gcnf* ^−/−^ embryos. (**A–B**) wt E8.5 embryos. (**C–D**) *Gcnf* ^−/−^ E8.5 embryos. (**E–F**) wt E9.5 embryos. (**G–H**) *Gcnf* ^−/−^ E9.5 embryos. Arrowheads indicate migratory PGCs. Note that the scale bars are 50 uM. Figures A, C, E and G are 25× and figures B, D, F, and H are 40×.

Two outstanding questions in the analysis of the *Gcnf*-mutant embryo phenotype are whether segregation of the germline has been compromised and whether Gcnf is required for further PGC development. To directly address these questions, we crossed the Oct4-GFP(ΔPE) reporter mice—a transgenic line generated with a construct lacking the proximal enhancer (PE) element of the *Oct4* promoter, which typically drives *Oct4* expression in the epiblast, here driving the expression of green fluorescent protein (GFP)—with our *Gcnf*-knockout (KO) mice. After gastrulation, widespread expression of Oct4 is repressed and only maintained in PGCs, which reside in the posterior of the embryo and allantois [22], [23]. Therefore, in this study the Oct4-GFP(ΔPE) reporter is specifically expressed in PGCs. We sacrificed pregnant female mice from a heterozygous cross at embryonic day (E) 8.5 and E9.5 (Figure 1). At E8.5, the embryos have not yet turned, and PGCs are clearly visible in the posterior of the embryo, as they begin their anterior migration (Figures 1A and 1B). In *Gcnf*-mutant embryos, morphological deformation is already evident, and PGCs can be observed in the posterior of the embryo; as there is an approximately equal number of PGCs in wild-type (wt) and mutant embryos (Figure 1C and 1D), Gcnf is not required for segregation of the germ cell lineage. At E9.5, the wt embryo has completed turning, and PGCs can be observed migrating anteriorly along the hindgut toward the developing midgut (Figures 1E and 1F). In contrast, *Gcnf*-mutant embryos fail to turn, and an ectopic tailbud forms due to posterior truncation. PGCs are still clearly visible in the posterior of mutant embryos, and rather than migrating along the hindgut, some are carried into the ectopic tailbud, indicating that Gcnf is not required for the maintenance of PGCs at this stage of embryonic development.

### Sexually dimorphic Gcnf expression in germ cells of the developing gonads

**Figure 2:**
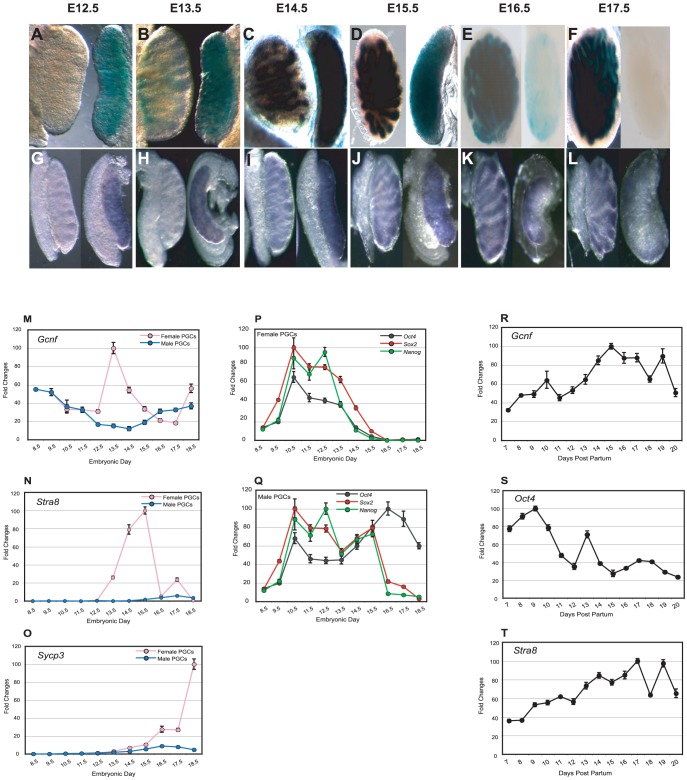
Analysis of *Gcnf*, pluripotency-associated genes, and meiosis-related gene expression profiles in PGCs in male and female embryonic gonads and in spermatogonial cells. (**A–F**) Analysis of β-galactosidase activity in male (left-hand side of each panel) and female (right-hand side of each panel) gonads in *Gcnf* LacZ KI embryos on E12.5 to E17.5. (**G–L**) WMISH analysis of *Gcnf* expression in the gonads of wt male (left-hand side of each panel) and female mice (right-hand side of each panel) on E12.5 to E17.5. (**M–Q**) Time-course analysis of gene expression profiles of PGCs from male and female genital ridges and embryonic gonads. Real-time q-RT-PCR analysis of (**M**) *Gcnf*. (**P–Q**) *Oct4, Nanog*, *and Sox2*. (**N**) *Stra8*, and (**O**) *Sycp3* in PGCs isolated by FACS sorting Oct4-GFP–positive cells from the genital ridges (E8.5 to E11.5) and from male and female fetal gonads on E12.5 to E18.5. (**R–T**) Time-course analysis of gene expression profiles of spermatogonial cells in newborn testes. Real-time q-RT-PCR analysis of (**R**) *Gcnf*. (**S**) *Oct4*, and (**T**) *Stra8* in spermatogonial cells isolated by FACS sorting Oct4-GFP–positive cells from the testes at 7 dpp to 20 dpp, i.e., during meiosis in male mice.

Although Gcnf is not required for germ lineage segregation or maintenance of PGCs during their migratory phase, it may play a role in later stages of germ cell development after formation of the gonads, when Oct4 is normally repressed and meiosis has been initiated. We generated a sensitive *Gcnf* reporter mouse, a *Gcnf* LacZ gene trap (GT) model [17]. We analyzed LacZ activity in the dissected gonads of male and female embryos from E12.5 to E17.5. In female gonads, *Gcnf* expression was detected on E12.5, maintained through E15.5, decreased by E16.5, and completely turned off by E17.5. In contrast, in male gonads, LacZ reporter activity was not detected until E13.5. β-Galactosidase activity continued to increase through E15.5 and was maintained through E17.5 (Figures 2A–2F). To ensure that the sexually dimorphic expression of *Gcnf* detected in the *Gcnf* LacZ Knockin (GT) mouse model reflected the expression of the normal gene, we analyzed *Gcnf* expression in wt mice by whole-mount *in situ* hybridization (WMISH). A very similar pattern of expression was observed. At E12.5 and E13.5, *Gcnf* was expressed in female, but not in male, gonads. By E14.5, *Gcnf* expression was detected in both gonads, but by E17.5, *Gcnf* expression was turned off in female gonads but maintained in male gonads (Figures 2G–2L).

To confirm that the *Gcnf* expression pattern observed in the male and female gonads is consistent with *Gcnf* expression in germ cells, we analyzed *Gcnf* expression in purified germ cells. Germ cells from gonads of Oct4-GFP(ΔPE) mice at different stages of development were isolated by fluorescence-activated cell storing (FACS) for GFP-positive cells. Expression of *Gcnf*, as well as meiosis-related and pluripotency-associated genes in female and male PGCs during fetal development was assessed by real-time quantitative RT-PCR (q-RT-PCR). In female PGCs, expression of pluripotency-associated genes, such as *Oct4*, *Sox2*, and *Nanog*, was steadily downregulated, whereas *Gcnf* expression was upregulated starting on E12.5, one day before the onset of meiosis (Figures 2M and 2P). In contrast, in male PGCs, which undergo mitotic arrest on E13.5, the mRNA levels of *Oct4* remained steadily high until birth (Figure 2Q), whereas expression of *Gcnf* and the meiosis-related genes *Stra8* and *Sycp3* remained low in all embryonic stages (Figures 2M, 2N, and 2O). To confirm the correlation of Gcnf and a regulatory role in meiosis, we determined the expression of *Gcnf*, *Oct4*, and the meiosis-related gene *Stra8* in spermatogonial cells isolated from testes of 7 days post partum (dpp) to 20 dpp. We observed a steady upregulation of expression of *Gcnf* and *Stra8* starting on 7 dpp, but a consistent downregulation of *Oct4* expression starting on 10 dpp, which is the onset of meiosis in the male mouse (Figures 2R–2T).

Taken together, these results show that *Gcnf* expression is upregulated, in contrast to *Oct4* expression, which is downregulated, in both female and male germ cells upon entry into meiosis. The temporal expression of *Gcnf* in male and female PGCs *in vivo* suggests a potential role for Gcnf in the downregulation of *Oct4* expression at the onset of meiosis and an involvement in either initiation of meiosis or activation of meiosis-related genes in both male and female germ cells.

### Knockdown of Gcnf *in vivo* impairs spermatogenesis

Next, we investigated the role played by Gcnf in meiosis. As adult unipotent germline stem cells (GSCs) derived from mouse testes can be expanded indefinitely in culture and can restore spermatogenesis after injection into the seminiferous tubules [24], [25], they are considered to be equivalent to spermatogonial stem cells *in vivo*. First, we found that *Gcnf* is expressed in GSCs (Figure S1). Then, we generated a *Gcnf*-knockdown model to monitor the effect of *Gcnf* knockdown during spermatogenesis. To this end, we infected GSCs with a lentiviral construct constitutively expressing the fluorescent marker Td-tomato and a shRNA targeting *Gcnf* (shGcnf) or *LacZ* (shLacZ) as a negative control. Two to 3 days after infection, more than 80% of the infected GSCs exhibited a positive tomato signal (Figures S2A, S2B, S4A, and S4B). Also we checked the efficiency of shGcnf and observed the downregulation of gene after 48 and 72 hours, which show 75% downregulation of *Gcnf* after 72 hours (Figure S4C). To determine the functionality of lentiviral-infected cells, GSCs containing shGcnf or shLacZ were transplanted into the seminiferous tubules of germ cell–depleted, busulfan-treated C57BL/6 mice. Only one testis per mouse was transplanted, with the non-transplanted testis serving as a control for endogenous spermatogenesis. Three months later, we observed colonization of the transplanted GSCs in both groups (Figures S2C–S2H).

**Figure 3:**
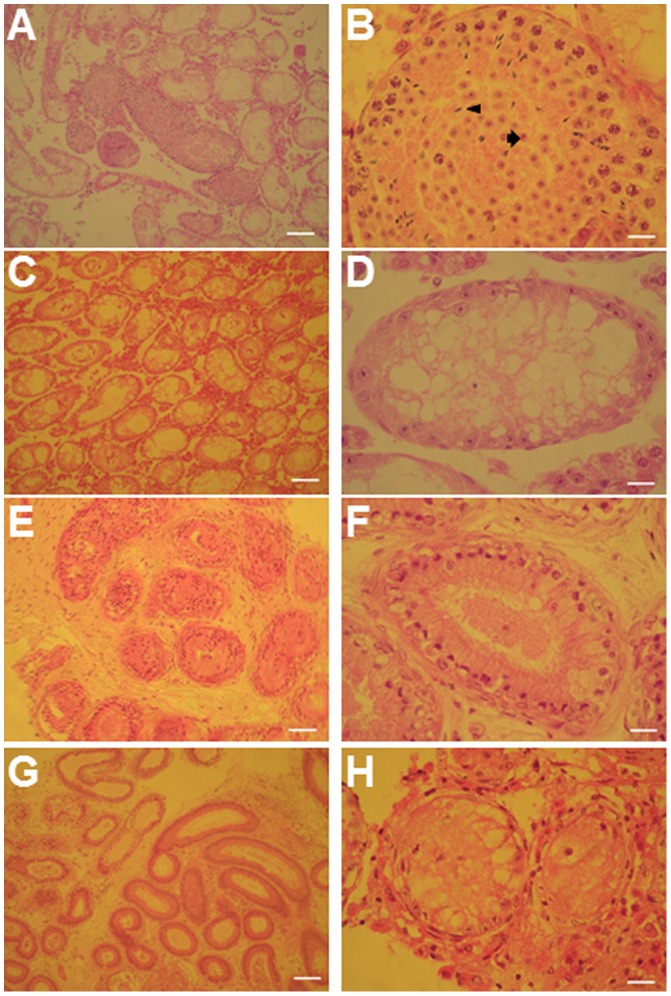
Histological sections of transplanted and non-transplanted GSCs into the testis. (**A–B**) GSCs without *Gcnf* siRNA (controls, only with tomato lentivirus). Note the presence of pachytene cells, round spermatids, and fully-grown sperm inside the testicular tubules of the right testis. (**C–D**) Non-transplanted testicular tubules of the left testis from the same mice, showing no recovery of germ cells in germ cell–depleted testes 3 months after treatment with busulfan. (**E–F**) GSCs with *Gcnf* siRNA. Note that GSCs colonized the testicular tubules of the right testis, but failed to differentiate into sperm. (**G–H**) Non-transplanted testicular tubules of the left testis from the same mice, showing no recovery of germ cells in germ cell–depleted testes 3 months after treatment with busulfan. Note that the scale bars in the left panel are 20 uM and in the right panel are 10 uM.

Furthermore, histological analysis of testicular sections of more than 10 transplanted testes from each group revealed that the transplanted shLacZ GSCs (control) had colonized the seminiferous tubules and restored spermatogenesis (Figures 3A and 3B). These tubules contained developing male germ cells of all stages, spermatids, and spermatozoa. In contrast, transplanted shGcnf GSCs had only colonized the tubules, but had failed to restore spermatogenesis (Figures 3E and 3F). Both groups of transplanted GSCs did not form teratomas in the host mice. In addition, histological sections of the non-transplanted testes from the same mice showed no restoration of spermatogenesis 3 months after transplantation, demonstrating that endogenous recovery of spermatogenesis had not occurred in germ cell–depleted mice (Figures 3C, 3D, 3G, and 3H). These results clearly demonstrate that knockdown of *Gcnf* blocks, or represses, further differentiation of GSCs into functional sperm within the seminiferous tubules of busulfan-treated mice.

### Impairment of the differentiation of Gcnf-deficient ESCs into PGCs *in vitro*

To assess the role played by GCNF in the formation of germ cells, we assessed the ability of *Gcnf*-deficient ESCs to generate PGCs *in vitro*. Wt and mutant *Gcnf* ESC lines were derived from *Gcnf* ^+/+^; Oct4-GFP(ΔPE) and *Gcnf* ^−/−^; Oct4-GFP(ΔPE) embryos from the same mouse model used in the studies in Figure 1. We analyzed the global gene expression patterns of PGCs derived *in vitro* on days 12 and 15 of differentiation of *Gcnf*-deficient ESCs (mutant) compared with those of *in vitro*–derived PGCs on days 12 and 15 of differentiation of Oct4-GFP(ΔPE) ESCs (wt). We had previously assessed the dynamics of gene expression and the formation of *in vitro*–derived PGCs from ESCs in a time-course analysis *in vitro*, and determined that cells on days 12 and 15 of differentiation exhibited the most PGC-like structural characteristics.

**Figure 4:**
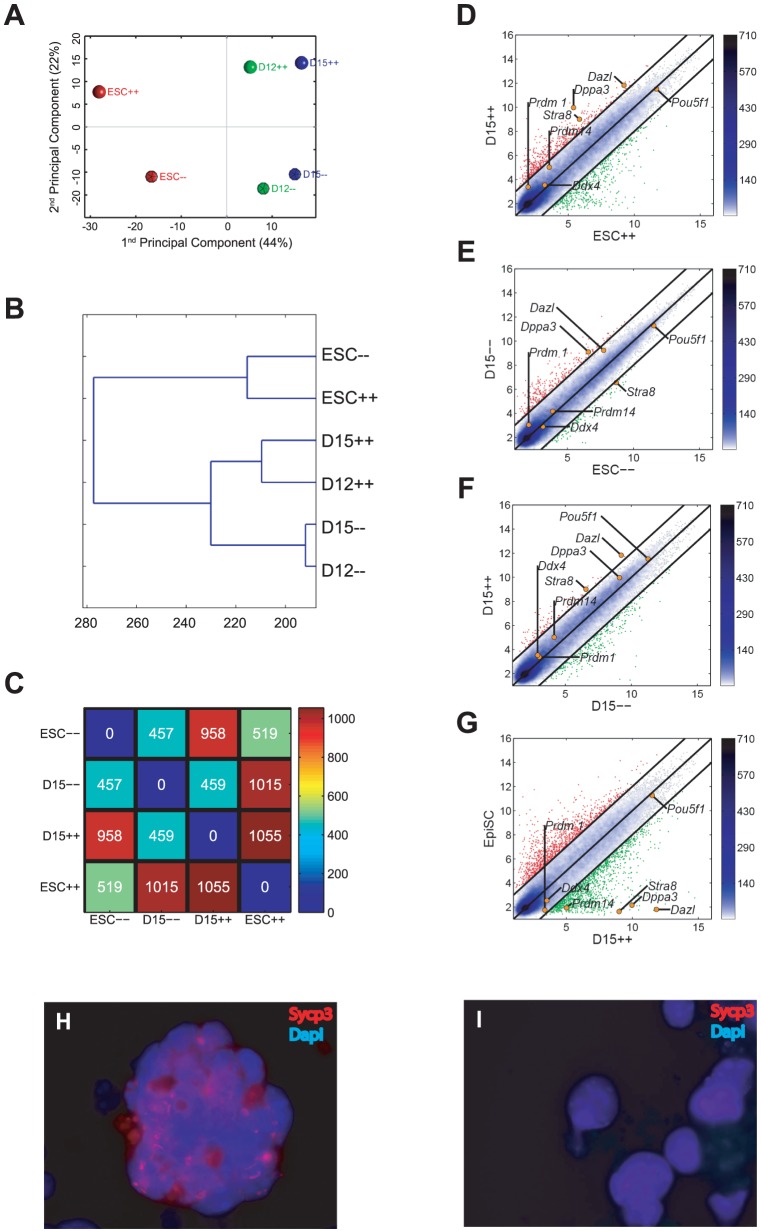
Global gene expression of Oct4-GFP(ΔPE) ESC-derived PGCs and *Gcnf*-deficient ESC-derived PGCs compared with wt ESCs. (**A**) Bi-dimensional PCA. The first principal component (PC1) captures 44% of the gene expression variability and the second principal component (PC2) captures 22%. (**B**) Hierarchical clustering. D denotes days, ++ denotes the wt, and − denotes the mutant groups. (**C**) Map of distances between samples (using as a metric the number of differentially expressed genes with fold change in log_2_ scale higher than two). The color bar to the right gives the color codification of the distances—the closer the two samples, the darker the blue color; the further the two samples, the darker the red color. (**D–G**) Pair-wise scatter plots of global gene expression in ESCs vs. *in vitro*–derived PGCs. Black lines indicate the boundaries of the two-fold changes in gene expression levels between the paired cell types. Color bar to the right indicates the scattering density—the higher the scattering density, the darker the blue color. Positions of some known PGC markers are shown as orange dots. Gene expression levels are log_2_ scaled. Genes upregulated in ordinate samples compared with abscissa samples are shown in red circles; genes downregulated are shown in green. (**H–I**) The localization of Sycp3 was performed in (**H**) Oct4-GFP(ΔPE) ESC-derived PGCs and (**I**) *Gcnf*-deficient ESC-derived PGCs of day-15 *in vitro*–differentiation cultures.

Principal component analysis (PCA) revealed that Oct4-GFP(ΔPE) ESC-derived PGCs exhibited a different global gene expression pattern compared with *Gcnf*-deficient ESC-derived PGCs on days 12 and 15 of differentiation *in vitro*. The second PCA component revealed a developmental difference between wt and *Gcnf*-deficient PGCs *in vitro* (Figure 4A). Hierarchical cluster analysis of the global gene expression pattern revealed that wt *in vitro*–derived PGCs clustered separately from *Gcnf*-deficient *in vitro*–derived PGCs (Figure 4B). A scatter plot of the global gene expression of ESCs and day-15 *in vitro*–derived PGCs (corresponding of final stage of *in vitro*–derived PGCs) showed a more dramatic increase in differentially expressed genes between ESCs and wt *in vitro*–derived PGCs than between ESCs and *Gcnf*-deficient *in vitro*–derived PGCs (Figures 4D and 4E). Specifically, ESCs differed from wt day-15 PGCs by 1,055 genes and from *Gcnf*-deficient day-15 PGCs by 457 genes (Figure 4C). In addition, a scatter plot of the global gene expression of wt and *Gcnf*-deficient PGCs derived on day 15 of *in vitro* differentiation showed that many genes were differentially expressed, particularly PGC-specific and meiosis-related genes (Figure 4F). Finally, comparison of the global gene expression pattern of wt day-15 *in vitro*–derived PGCs with epiblast stem cells (EpiSCs) showed a dramatic number of differentially expressed genes, thus demonstrating proper germ cell differentiation *in vitro* (Figure 4G). These results suggest that in the absence of Gcnf, the derivation of *in vitro* PGCs is impaired.

To confirm these findings, we determined the expression of day-15 *in vitro*–derived PGCs by real-time q-RT-PCR. Expression of all known germ cell and meiotic markers was found to be upregulated to moderate levels in Oct4-GFP(ΔPE) ESC-derived PGCs compared with Oct4-GFP(ΔPE) ESCs, whereas the expression of these genes was upregulated to a lesser extent in *Gcnf*-deficient ESC-derived PGCs compared with *Gcnf*-deficient ESCs (Figures S3A and S3B). We identified 11 genes that were expressed in PGCs but not in pluripotent stem cells [26]. The majority of these genes were found to be expressed in wt day-15 *in vitro*–derived PGCs but not in day-15 *Gcnf-deficient* ESC-derived PGCs (Figures S3C and S3D). We examined the localization of synaptonemal complex protein 3 (Sycp3) in both groups of *in vitro*–derived PGCs on day 15 of differentiation, and showed the positive immunostaining for this protein in Oct4-GFP(ΔPE) ESC-derived PGCs but not in *Gcnf*-deficient ESC-derived PGCs (Figures 4H and 4I).

To exclude the possibility that day-15 PGCs are functionally pluripotent, we subcutaneously injected both wt and mutant day-15 *in vitro*–derived PGCs into severe combined immunodeficiency (SCID) recipients. One month after the injection, we did not observe any teratomas *in vivo*, further confirming that these cells were not pluripotent ESCs. Taken together, our observations suggest that Gcnf is essential for the differentiation of PGCs and initiation of meiosis *in vitro*.

### Gcnf is required for development of female fetal PGCs into oocytes

**Figure 5:**
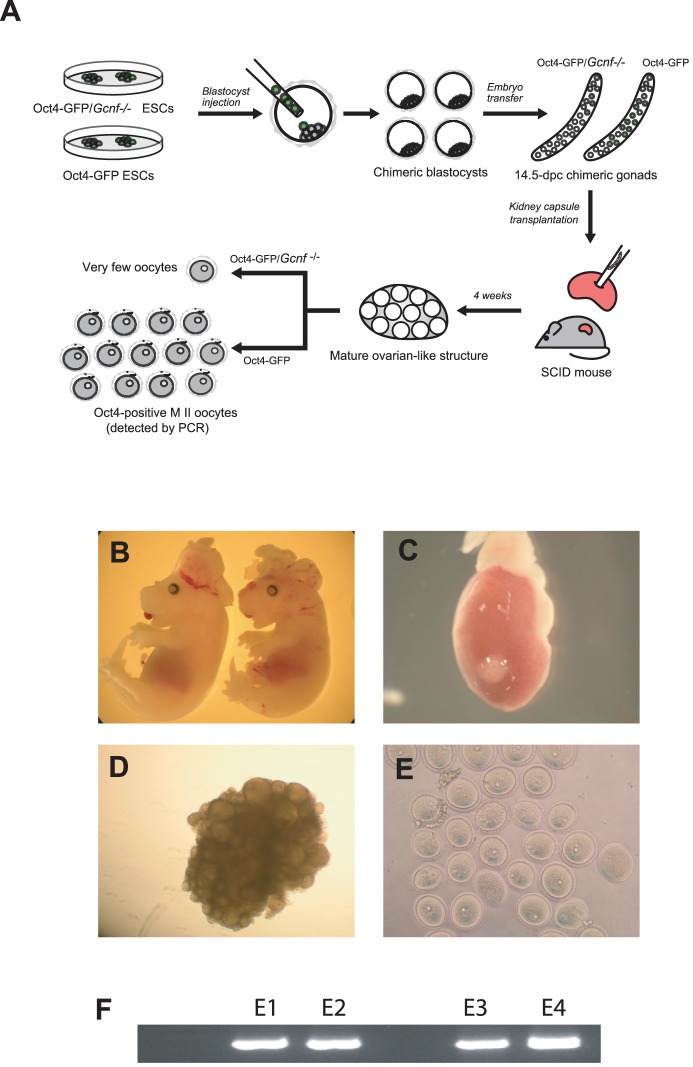
(**A**) Schematic overview of blastocyst injection, germline contribution, and kidney capsule transplantation of Oct4-GFP ESCs and *Gcnf* ^−/−^; Oct4-GFP(ΔPE) ESCs. (**B**) E14.5-chimeric *Gcnf* ^−/−^ embryos; note that the neural tubes and brain are affected. (**C–D**) E14.5-chimeric female gonads developed an ovarian-like structure under the kidney capsule of SCID recipients 4 weeks after transplantation. (**E**) MII oocytes isolated from only the wt ovarian-like structure and (**F**) expression of GFP performed by nested PCR from an isolated MII oocyte (E1 to E4 depicts the experimental replicate numbers).

To examine whether *Gcnf*-deficient ESCs have the developmental potential to differentiate into oocytes *in vivo*, we used a blastocyst injection strategy with XX Oct4-GFP(ΔPE) ESCs and XX *Gcnf* ^−/−^; Oct4-GFP(ΔPE) ESCs (Figure 5A) to generate chimeras. We then transferred the injected blastocysts into pseudopregnant recipients and subsequently observed germline contribution in the gonads of an E14.5 embryo by means of Oct4-GFP expression. The germline contribution of *Gcnf* ^−/−^; Oct4-GFP(ΔPE) ESCs was estimated to be 15% compared with 45% for control ESCs (wt Oct4-GFP(ΔPE) ESCs), a difference of more than 60%. As more than 30% of chimeric embryos generated from *Gcnf*-deficient ESCs have brain and neural tube deficiency owing to the involvement of Gcnf in neurogenesis [9], [19] (Figure 5B), we rescued female chimeric gonads by transplanting them under the kidney capsule of SCID mice and examined them after 4 weeks to determine whether wt and *Gcnf* ^−/−^ chimeric gonads had the same potential to develop from early meiotic germ cells into mature oocytes. We observed that both groups of PGCs could develop ectopically into an ovarian-like structure (54% for control ESCs compared with 51% for *Gcnf* ^−/−^ ESCs) under the kidney capsule (Figures 5C and 5D), but we were only able to isolate fully developed MII oocytes from the ovarian-like structures of chimeric gonads generated by blastocyst injection with Oct4-GFP(ΔPE) ESCs (35–40 MII oocytes from each ovarian-like structure) (Figure 5E). We did not observe fully developed oocytes from the ovarian-like structures of *Gcnf* ^−/−^ chimeric gonads, but only a few degenerated oocytes (5–10 degenerated oocytes from each ovarian-like structure), confirming that *Gcnf*-deficient ESCs have no developmental potential and differentiate into oocytes *in vivo*. Although, for unknown reasons, we could not monitor GFP signal in fully developed oocytes under the fluorescence microscope, we were able to detect *GFP* expression by nested PCR in cumulus cell–free oocytes derived from control ESCs (Figure 5F).

### Gcnf is required for the repression of *Oct4* expression in embryonic gonads

**Figure 6:**
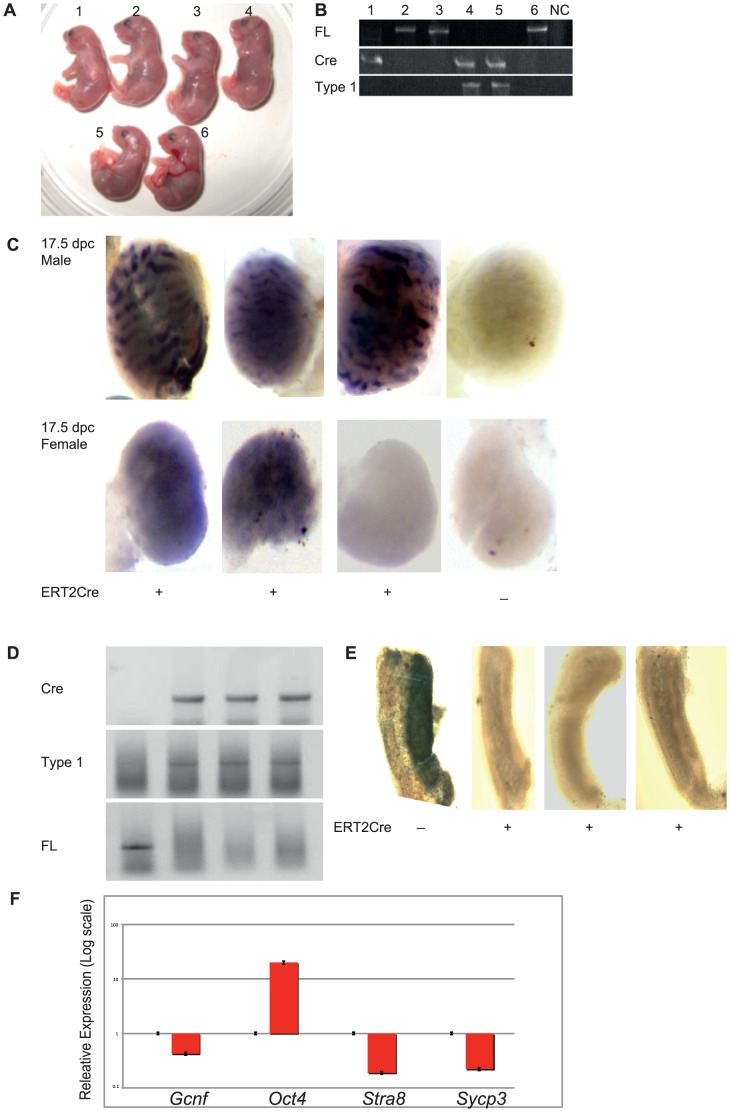
Analysis of the role of Gcnf and Oct4 in gonadal development in Cre/Lox mutation of the *Gcnf* gene. (**A–B**) The genotyping determined whether the embryos were Cre positive or negative, and the status of the floxed *Gcnf* allele. (**C**) WMISH analysis of *Oct4* expression on E17.5 in male and female gonads of control *Gcnf* wt (Cre−) treated with 4-OH tamoxifen and 4-OH tamoxifen–induced ERT2Cre-inactivated *Gcnf^−/−^* (Cre+) embryos. (**D**) The status of the floxed *Gcnf* allele. (**E**) WMISH analysis of *Stra8* expression on E15.5 in female gonads of control *Gcnf* wt (Cre−) treated with 4-OH tamoxifen and 4-OH tamoxifen–induced ERT2Cre-inactivated *Gcnf^−/−^* (Cre+) embryos. (**F**) Real-time q-RT-PCR analysis showed that two days of treatment with doxycycline resulted in the upregulation of Oct4 expression and the downregulation of *Stra8* and *Sycp3* expression in 14.5-dpc female gonads of Tet-Oct4 pregnant mice (14.5-dpc female PGCs were considered as 1 for normalization).

During embryonic development, *Oct4* expression is regulated in a sexually dimorphic manner. In female gonads, the expression of *Oct4* is silenced upon entry of cells into meiosis. However, in male gonads, *Oct4* expression is also repressed later on E17.5. The inverse correlation between *Gcnf* and *Oct4* expression in developing gonads suggests that the retinoid-induced repression of *Oct4* during the differentiation of ESCs may reflect a role for Gcnf in the repression of *Oct4* expression during gonadal development [6]. To test this hypothesis *in vivo*, we generated a regulated KO of *Gcnf* by combining *Gcnf* ^fl/fl^ mouse model with an ERT2Cre mouse model [27] in which this regulator had been knocked into the ROSA locus. We treated pregnant female mice with 4-OH tamoxifen on E11.5 (a time point late enough not to affect the early developmental requirement for GCNF) and then harvested embryos on E17.5 to analyze *Oct4* expression. The experiment was designed such that each embryo would be homozygous for the floxed allele and be either Cre positive or negative. The Cre-negative embryo would serve as control for tamoxifen treatment, which can slow embryonic development. An E17.5 litter was weighed, the gonads were dissected, and DNA was isolated from the remainder of the embryos for genotyping. The genotyping identified the embryos as Cre positive or negative and revealed the status of the floxed *Gcnf* allele (Figures 6A and 6B). In the Cre-negative embryos, the *Gcnf* floxed allele was intact, while in Cre-positive littermates, the floxed allele was completely recombined to yield what we term a Type I deletion, a deletion in the DNA binding domain–encoding exon [3] (Figure 6B).

WMISH analysis of *Oct4* expression in the male gonads on E17.5 showed that Cre-negative embryos exhibited low *Oct4* expression, while Cre-positive embryos showed loss of repression of *Oct4* expression (10 of 10 embryos). In female embryos, two events were observed. In some embryos, there was repression of *Oct4* expression on E17.5, while in others, there was clear loss of repression of *Oct4* expression on E17.5 (Figures 6C). The difference between the two types of female responses is most likely due to the timing of Cre recombination of the floxed *Gcnf* alleles. In female gonads with abrogated *Oct4* repression, the *Gcnf* allele is inactivated at an early enough time point to prevent significant *Gcnf* expression. In female gonads with silenced *Oct4* expression, the *Gcnf* gene is not completely inactivated, resulting in induction of *Gcnf* expression and production of enough Gcnf protein to silence *Oct4* expression. However, Gcnf is clearly required to repress *Oct4* expression during the critical stages of gonadal development.

To confirm the correlation of Gcnf and Oct4 expression in gonadal development and particularly the requirement for *Oct4* levels in the initiation of meiosis, we first performed conditional ectopic overexpression of *Oct4* levels in 14.5-dpc female gonads. To assess the short-term effect of *Oct4* ectopic expression *in vivo*, we crossed a Tet-Oct4 mouse [28] with an Oct4-GFP mouse. We fed pregnant female mice drinking water that contained doxycycline on E12.5 (a time point when meiosis-related genes just start to be overexpressed in developing female gonads) and then harvested the embryos on E14.5 to analyze *Oct4*, *Stra8*, and *Sycp3* expression.

Interestingly, ectopic activation of *Oct4* in developing female gonads results in the inhibition of expression of meiosis markers such as *Stra8* and *Sycp3* (Figure 6F) and the interruption of meiotic initiation as typically seen in female gonads at this time of PGC development (Figures 2N and 2O). Taken together, these results showed that downregulation of *Oct4* expression is clearly required to activate *Stra8* and *Sycp3* expression during the onset of meiosis in developing female gonads, and confirmed that meiosis markers are not upregulated in developing male germ cells before birth, as *Oct4* levels are not reduced in male gonads.

### Gcnf is required for enhancing *Stra8* expression in embryonic gonads

*Stra8* expression is regulated in a sexually dimorphic manner during embryonic development. In female gonads, the expression of *Stra8* is activated upon entry of cells into meiosis on E12.5, increases gradually till E15.5, and is dramatically downregulated on E16.5 at prophase I arrest in female gonads (Figure 2N). However, in male gonads, *Stra8* expression is nearly completely repressed during embryonic development. In male gonads, *Stra8* expression is activated in the testis during neonatal development, increases steadily starting on 7 dpp, and continues till 19 dpp, coincident with the onset of meiosis in male spermatogonial cells (Figure 2T). The direct correlation between *Gcnf* and *Stra8* expression in developing gonads (Figure 2M, 2N and 2T) suggests that the retinoid-induced activation of *Stra8* expression during the differentiation of ESCs may reflect a role for Gcnf in the activation of *Stra8* expression during gonadal development [7], [29]. To test this hypothesis *in vivo*, we used the same KO model for *Gcnf* by crossing the *Gcnf* ^fl/fl^ mouse with an ERT2Cre mouse. We treated pregnant female mice with 4-OH tamoxifen on E11.5 (a time point late enough not to affect the early developmental requirement for Gcnf) and then harvested embryos on E15.5 to analyze *Stra8* expression.

WMISH analysis of *Stra8* expression in the female gonads on E15.5 showed that Cre-negative embryos exhibited high *Stra8* expression, while Cre-positive embryos showed loss of activation of *Stra8* expression (4 of 4 embryos) (Figure 6D–6E). This means that in female gonads with loss of *Gcnf* expression, the *Stra8* gene is not completely activated, resulting in inhibition of meiotic initiation. These results showed that Gcnf is clearly required to activate *Stra8* expression during onset of meiosis in the development of both male and female gonads.

## Discussion

Previous studies [1], [10], [11], [30]–[32] have shown that the *Gcnf* gene is active in adult testes and ovaries and is required for reproduction in female mice [17]. In adult testis, Gcnf acts as a transcriptional regulator of the postmeiotic genes Protamine 1 and 2 (*Prm 1, Prm 2*), functionally inhibiting CREMtau [4], [13], [14], [33], whereas in the adult ovary, Gcnf regulates the expression of bone morphogenetic protein 15 (*Bmp15*) and growth differentiation factor 9 (*Gdf9*) [17]. The Cre/Lox mutation of floxed *Gcnf* by ZP3Cre leads to subfertility in female mice [17]. Our current study demonstrates that Gcnf is involved in male and female gametogenesis during mouse development. We show that loss of Gcnf results in failure to produce spermatogonia and oocytes in developing testes and ovaries, respectively. Our study also shows that Gcnf is required to repress *Oct4* expression during gonadal development.

Segregation of germ cells in the mouse occurs on E7.25 of embryonic development, when PGCs arise as a small cluster of alkaline phosphate–positive cells in the extraembryonic mesoderm [34]–[38]. At this developmental time point, Gcnf is functionally active and is required to repress pluripotency genes such as *Oct4*—as the pluripotent late epiblast is patterned during gastrulation—and *Gcnf* ^−/−^ embryos begin displaying phenotypic alterations compared with wt embryos. Thus, to determine whether Gcnf is required in the formation of the PGC lineage, we crossed the PGC reporter Oct4-GFP(ΔPE) with *Gcnf* ^−/−^ mice. We clearly observe GFP-positive PGCs in the posterior of *Gcnf* ^−/−^ embryos. Thus, Gcnf is not required for the segregation of the germ cell lineage. The normal anterior migration of PGCs from the allantois to the midgut, however, is disrupted in the Gcnf^−/−^ embryos, and some PGCs even migrate into the ectopic tail bud [8]. Although we cannot rule out that Gcnf plays a direct role in the migration and homing of PGCs, the defect is more likely a secondary effect of the developmental defects of KO embryos.

To determine whether Gcnf plays a role in later stages of PGC development, we analyzed *Gcnf* expression at various stages of germ cell development. *Gcnf* LacZ Knockin (KI) mice displayed sexually dimorphic expression of β-galactosidase activity, suggesting that Gcnf plays a role in gonadal development. To confirm that this reporter accurately reflected *Gcnf* expression, we performed WMISH hybridization, which confirmed the sexually dimorphic expression observed. We then isolated germ cells from transgenic mice harboring the Oct4-GFP(ΔPE) reporter to confirm that the expression of *Gcnf* was indeed confined to the germ cell compartment. In addition, we analyzed the expression of meiosis-related genes as well as pluripotency-associated genes, as *Oct4* is the main pluripotency-related gene required for the maintenance and survival of PGCs [21].

In female embryonic gonads, upregulation of *Gcnf* expression begins at around E12.5, concordant with the upregulation of the premeiosis-related gene *Stra8*. In contrast, downregulation of pluripotency-associated genes, such as *Oct4*, *Sox2*, and *Nanog*, is observed at the onset of meiosis. Gene expression analysis in spermatogonial cells of newborn testis and embryonic female gonads confirmed a correlation between *Gcnf*, *Oct4*, and *Stra8* expression at the onset of meiosis in both sexes. In contrast, we did not observe coincident upregulation of *Gcnf* with *Stra8* and *Sycp3* expression, and downregulation of *Oct4*, *Sox2*, and *Nanog* expression in male embryonic gonads at mitotic arrest on E13.5. As Gcnf represses the expression of *Oct4* [2], these results suggest a possible association between Gcnf, Oct4, and Stra8 in regulating the initiation of meiosis in germ cells. To confirm this hypothesis and the requirement for *Oct4* downregulation in the activation of *Stra8* and *Sycp3* expression during onset of meiosis in the development of female gonads, we demonstrated that upregulation of *Oct4* levels alone inhibited the expression of meiosis markers such as *Stra8* and *Sycp3* and consequently interrupted meiotic initiation. This suggests that the concomitant activation of Gcnf expression and Oct4 repression is required for the activation of meiosis-related genes.

We previously reported that adult unipotent germline stem cells derived from mouse testes could colonize the seminiferous tubules of busulfan-treated mice and restore spermatogenesis [24]. Here, we observed that knockdown of *Gcnf* by using small hairpin RNA (shRNA) in GSCs inhibits the differentiation of GSCs into functional sperm and blocks the restoration of spermatogenesis after transplantation of these cells into the seminiferous tubules of busulfan-treated mice. This observation is consistent with those of previous reports [1], [10], [11], [30]–[32], confirming a functional role for Gcnf in spermatogenesis in the mouse.

To support a potential role for Gcnf during germ cell development and onset of meiosis, we assessed the ability of *Gcnf*-deficient ESCs to differentiate into PGCs *in vitro*. By using global gene expression analysis, real-time q-RT-PCR of known and newly identified PGCs markers, and localization of meiosis-related proteins, we showed that in contrast to wt ESCs, *Gcnf* ^−/−^ ESCs fail to differentiate into PGCs and initiate meiosis *in vitro*. Previous studies have shown that *Gcnf* is expressed in developing oocytes during folliculogenesis in the mouse [1], [10], [11]. Gcnf may be involved in female fertility by regulating the expression of *Bmp15* and *Gdf9* [17]. In this study, we demonstrated that Gcnf is required for the development of female fetal PGCs into oocytes. Although both wt and *Gcnf*-deficient ESCs were able to contribute to the germline, following the transplantation of a chimeric fetal ovary under the kidney capsule of SCID mice, *Gcnf* ^−/−^ PGCs could not generate mature oocytes inside the grafted ovary. Our finding suggests that Gcnf is essential for further development and maturation of PGCs into oocytes.

Here we report that Gcnf is required for spermatogenesis and oogenesis in the mouse and that loss of Gcnf disrupts the formation of functional gametes. Taken together, our findings using (1) gene expression in PGC development, (2) disruption of spermatogenesis after downregulation of *Gcnf*, (3) impairment of *in vitro* differentiation of PGCs by using *Gcnf* ^−/−^ ESCs, and (4) the inability of ectopic development of a chimeric fetal ovary under the kidney capsule of host mice all provide a robust model for a regulatory role for Gcnf in germ cell development. Support for the role of Gcnf in the maintenance of germ cells has been provided by *ex vivo* experiments using an *in vivo*–regulated Cre/Lox KO of *Gcnf* during gestation. The *Gcnf* gene was successfully deleted in the gonads and the embryo as a whole, and defects in *Oct4* expression were observed. These defects are due to inactivation of the *Gcnf* gene in PGCs, as opposed to a secondary effect on development, as the whole embryos were normal overall. These results contrast those of a similar KO strategy by the Page group, who reported no functional requirement for Gcnf or its ligand-binding domain in germ cell development [39]. The two Gcnf floxed models differ in the regions deleted; in addition, another significant difference between the two studies is the use of tamoxifen to activate ERT2Cre, which first has to be metabolized into the active form 4-hydroxy tamoxifen before it can activate ERT2Cre, which would cause a functional delay to generate defects in *Oct4* expression. We used 4-hydoxy tamoxifen to activate ERT2Cre, which would allow for earlier deletion of Gcnf expression and thus readily generate a phenotype.

Clearly, like in ESCs, Gcnf plays an essential role in the repression of *Oct4* expression in female gonads as they exit mitosis and enter meiotic prophase I. Postnatally, Gcnf likely plays a role in maintaining germ cells in both male and female newborns, as shown in the various experiments detailed here. This functional window must lie between the ERT2Cre KO of *Gcnf* during embryonic development, as the gonads still contain germ cells *and* the late-stage ZP3Cre KO of *Gcnf* in adult female mice, which showed fertility defects but no loss of germ cells. Other mouse models are needed to delineate this important function of Gcnf in early postnatal life.

Several research groups have shown that the *Stra8* gene (stimulated by retinoic acid gene 8) is a premeiotic marker that is expressed in premeiotic germ cells of the testes and ovaries and is regulated by retinoic acid [40], [41]. Loss of *Stra8* gene function in germ cells leads to inhibition of onset of meiosis and results in the failure of germ cells to undergo meiosis in the ovaries or testes of *Stra8* ^−/−^ mice [42]–[44].

We hypothesize that Gcnf plays either a direct or indirect role in germ cell development, and suggest a possible function for Gcnf in the activation or initiation of meiosis by enhancing *Stra8* expression and inhibiting the expression of pluripotency-associated genes. This knowledge should enhance our understanding of the key mechanisms underlying germ cell development and onset of meiosis as well as prove beneficial for the derivation of viable and fertile sperm and oocytes from pluripotent stem cells *in vitro*.

## Methods

### Animal ethics

This study was performed in accordance with the recommendations of the Federation of Laboratory Animal Science Associations (FELASA). The corresponding ethics protocol for mice works was approved by the Landesamt für Natur, Umwelt und Verbraucherschutz (LANUV) of the state of North Rhine-Westphalia in Germany, and the corresponding ethics protocol for testicular transplantation was approved by Center for Stem Cell Research, Institute of Biomedical Sciences and Technology, Konkuk University, Seoul, Republic of Korea.

### Analysis of the Oct4-GFP(ΔPE) in Gcnf KO embryos

Transgenic mice harboring the Oct4-GFP(ΔPE) reporter were crossed with *Gcnf* ^+/−^ mice to generate *Gcnf* ^+/−^; Oct4-GFP(ΔPE) mice, which were subsequently crossed with *Gcnf* ^+/−^ mice to generate embryonic litters with +/+, +/−, and −/−. Timed pregnant female mice were euthanized and embryos were harvested on E8.5 and E9.5. The fluorescent Oct4-GFP(ΔPE) reporter was visualized in PGCs using a Zeiss Stemi SV11.

### Reporter LacZ staining

LacZ staining of embryonic gonads, which were isolated from matings between *Gcnf* LacZ KI heterozygous mice on E12.5–E17.5 with X-Gal staining, was performed according to the manufacturer's protocol (Millipore, Billerica, MA). The yolk sacs of the embryos were lysed for genotyping.

### WMISH

*Gcnf* ^fl/fl^ mice were crossed with ROSA ERT2Cre mice to generate *Gcnf* ^fl/+^ ERT2Cre mice, which were then crossed with *Gcnf* ^fl/fl^ mice; female mice were checked daily for plugs and treated with 1 mg 4-OH tamoxifen (Sigma, H7904) once on E11.5. The gonads were isolated from embryos on E17.5 and fixed in 4% paraformaldehyde. The yolk sacs of embryos were lysed for genotyping. WMISHs were carried out as described previously [2]. Genotyping Primers:

*Gcnf* LacZ KI:

Primer1: 5′TCGAGCGATGTTTCGCTT3′;

Primer2: 5′ATATGGGATCGGCCATTGA3′;

Primer3: 5′CAGTGCTGACTTATCCATG3′;

Primer4: 5′TTCCTGTTCATGCCCATCT3′.

*Gcnf* fl:

PCR-A: 5′CGAACTCAGAAATCCACC3′;

PCR-C: 5′AATCACAAACACCACAACTC3′.

*Gcnf* Type I:

Type I-F: 5′CCAATTCCCCCAAAGTGTC3′

Type I-R: 5′CAGGTCGAGGGACCTATAAC3′.

ERT2Cre:

Cre-F: 5′CGGTCGATGCAACGAGTGAT3′;

Cre-R: 5′CCACCGTCAGTACGTGAGAT3′.

### Isolation of PGCs from mouse fetus and flow cytometric analysis

Germ cells of E8.5 to E18.5 embryos were isolated from OG2 (Oct4ΔPE-GFP)/CD1 mice by disaggregating the genital ridges of E8.5 to E11.5 embryos and the fetal gonads of E12.5 to E18.5 embryos. Disaggregation was performed by enzymatic digestion with collagenase type 1A (Sigma) and trypsin-EDTA (0.25%) at a ratio of 1∶2 with shaking at 700 rpm for 8 min at 37°C. Single-cell suspension was achieved more efficiently by triturating the cell aggregates. Enzymatic digestion was stopped by adding a two-fold excess of DMEM/F12+10% FBS. Cell suspensions were washed once in DMEM/F12+10% FBS and FACS sorted for GFP. GFP-positive PGCs were directly sorted into lysis buffer RLT (QIAGEN, Hilden, Germany) using a FACSAria cell sorter (BD Biosciences). Single-cell suspensions of GFP-positive PGCs were analyzed using a FACSAria cell sorter (BD Biosciences). Data analysis was done using FlowJo software (Treestar).

### RNA isolation

RNA from FACS-sorted, GFP-positive PGCs was isolated using the RNeasy micro and mini kits (QIAGEN) with on-column DNase digestion as per the manufacturer's instructions. The quality of RNA was determined using a Nanodrop (ND-1000) spectrophotometer and an Agilent RNA 2100 Bioanalyzer.

### RT-PCR and real-time quantitative RT-PCR

Total RNA (250 ng) was reverse transcribed into cDNA by using the High-Capacity cDNA Reverse Transcription Kit (Applied Biosystems, Darmstadt, Germany). q-RT-PCR was performed on 7900ht and ht fast devices (Applied Biosystems) with milliQ H_2_O and TaqMan Universal PCR Master Mix (Applied Biosystems). Raw data was obtained by SDS 2.3 (Applied Biosystems). Gene expression was normalized to the mouse housekeeping gene *Hprt*. Relative quantitation of gene expression was calculated using the ΔΔCt method. Three technical replicates were used for each real-time q-RT-PCR reaction; a reverse transcriptase blank and a no-template blank served as negative controls. Primer sequences for real-time q-RT-PCR are listed as supplementary material (Tables S1 and S2).

### Vector construction and transduction with lentiviral vectors

pLVTHM-Td tomato was constructed by replacing the GFP in pLVTHM with the Td-tomato coding sequence. DNA constructs designed to produce shRNA hairpins targeting Gcnf (5′- GGATGAATTGGCAGAGCTTGATTCAAGAGATCAAGCTCTGCCAATTCATCC –3′) or LacZ (5′-GTGGATCAGTCGCTGATTAAATTCAAGAGATTTAATCAGCGACTGATCCAC –3′) were cloned in front of the H1 promoter into the pLVTHM-Td tomato vector to produce pLVTHM-shGcnf and pLVTHM-shLacZ, respectively. The 293T cell line was cultured in DMEM supplemented with 15% FBS. The recombinant lentiviruses were produced by transient transfection of 293T cells with 12 µg of pLVTHM-shGcnf or pLVTHM-shLacZ, 8.5 µg of psPax2, and 3 µg of pMD2.G using Lipofectamine 2000 (Invitrogen) according to the manufacturer's protocol. The supernatant was collected at 24 and 48 hr of transfection and concentrated by ultracentrifugation at 26,000 rpm for 2 hr at 4°C using an SW41 rotor (Beckman Coulter). After ultracentrifugation, the supernatant was decanted, and the viral pellet was resuspended in 200 µl of DMEM. The suspension was stored at −80°C until use. pLVTHM and packaging plasmids were provided by D. Trono (Geneva, Switzerland). GSCs were plated on 24-well plates (5×10^4^ cells/well), and 20 µl of the concentrated virus were added to the medium. Cells were washed after 16 hr of incubation.

### Testicular transplantation

The transplantation experiments were performed as previously described [45]. Four-week-old germ-cell–depleted (40 mg/kg busulfan-treated) male mice were used as recipient mice. Approximately 3×10**^5^** cells were injected with a micropipette (80-µm diameter tips) into the seminiferous tubules of the testes of recipient mice through the efferent duct. Three months after the transplantation, the mice were sacrificed, and the seminiferous tubules were dissociated by collagenase treatment and examined under a fluorescence microscope to detect transplanted GSCs by their RFP expression. To assess further colonization as well as restoration of spermatogenesis, the testes were fixed in Bouin's solution and embedded in paraffin. Sections were stained with hematoxylin and eosin (H&E).

### ESCs

Male and female Oct4-GFP(ΔPE) ESCs and female *Gcnf* ^−/−^ ESCs [8] were grown on gamma-irradiated mouse embryonic fibroblasts (MEFs) in KNOCKOUT DMEM medium containing 4.5 g/l glucose and supplemented with 15% KNOCKOUT SR (Invitrogen), 2 mM L-glutamine (Invitrogen), 100 µM nonessential amino acids (Invitrogen), 1 µM 2-mercaptoethanol (Invitrogen), and 50 µg/ml each penicillin and streptomycin (Invitrogen) in the presence of 1,000 U/ml murine leukemia inhibitory factor (LIF) (ESGRO; Chemicon).

### *In vitro* differentiation of PGCs from ESCs

Oct4-GFP(ΔPE) ESCs and *Gcnf* ^−/−^ ESCs were grown in 0.1% gelatin-coated tissue culture plates in ESC medium supplemented with 15% FBS (Invitrogen) but without LIF and in the absence of MEFs at a density of approximately 8–9×10**^4^** cells/cm**^2^**. Non-adherent cells were removed after 3 days and the medium was replaced. GFP expression was monitored every 3 days in cells undergoing further differentiation until day 15. Approximately 25% of all cells exhibited GFP expression on day 15. The medium in the master culture plates was replaced every 2 days [46].

### Isolation of *in vitro*–derived PGCs from differentiation culture plates

Every 3 days, cells were dissociated with 0.25% trypsin-EDTA, neutralized with KNOCKOUT DMEM containing 15% FBS, washed twice with PBS, and then resuspended in KNOCKOUT DMEM containing 15% FBS. Approximately 2×10^6^ cells/ml in DMEM/FBS were used for FACS sorting. Flow cytometry was performed on a FACSAria cell sorter. Putative PGCs derived from ESCs were sorted based on *Oct4*-GFP expression and then subjected to germ cell characterization, e.g., gene expression, ability for tumor formation, and localization of meiosis-related proteins.

### Teratoma formation

Day-12 and day-15 *in vitro*–derived PGCs were suspended in DMEM containing 15% FBS. A 200-µl aliquot (1×10^6^ cells) of the cell suspension was injected subcutaneously into the dorsal flanks of SCID mice. Following injection of the *in vitro*–derived PGCs, wt and mutant ESCs were also subcutaneously injected into the SCID mice as controls. Four weeks after the injection, tumors were dissected from the mice. Samples were fixed in PBS containing 4% paraformaldehyde and embedded in paraffin. Tissue sections were stained with H&E.

### Immunohistochemistry

Immunohistochemistry for Sycp3 was performed using the spreading technique, as previously described by others [47]. Slides were incubated with primary anti-Sycp3 (1∶500; Abcam) for 2 hr at room temperature. After washing in blocking solution, slides were incubated with secondary fluorescent antibody (1∶1,000; Alexa 568; Molecular Probes) for 1 hr at room temperature and then mounted in DAPI-containing mounting medium (Vectashield; Vector Laboratories Inc.).

### Chimera formation

Six- to eight-week-old female mice (B6C3F1) were induced to superovulate (via intraperitoneal injection of 7.5 I.U. PMSG and 48 hr later of 7.5 I.U. hCG) and mated with CD1 male mice. Blastocysts were collected on day 3.5 after a vaginal plug check and flushed in HCZB medium containing 0.1% polyvinylpyrrolidone (PVP). Blastocysts were washed extensively with HCZB medium and cultured in Alpha MEM medium containing 0.2% BSA at 37°C, 5% CO_2_ in air until injection with ESCs.

Forty to sixty ESC colonies were selected and picked under a stereomicroscope based on the colony shape and morphology, washed with PBS, and then transferred into a drop of 0.05% trypsin-EDTA to obtain a single-cell suspension. Single cells were then transferred into the micromanipulation chamber in a drop of HCZB medium containing 0.1% PVP and 0.2% BSA. Groups of 12–15 cells were injected into a single blastocyst. Injected embryos were then transferred into a drop of Alpha MEM-BSA and cultured at 37°C, 5% CO_2_ in air. After 4 hr of culture, chimeric blastocysts were transferred into the uterine horns of 2.5-dpc pseudopregnant CD1 recipient female mice.

### Transplantation of chimeric ovaries under the kidney capsule

Fourteen days after transfer of the chimeric blastocysts into the uterine horns, the fetal ovaries of 14.5-dpc embryos were obtained from pregnant pseudopregnant CD1 recipient female mice and screened for germline contribution under fluorescent microscope. One to two chimeric fetal ovaries without attached mesonephroses were transplanted under the kidney capsule of ovariectomized female SCID recipient mice. Four weeks after transplantation, chimeric ovarian grafts were removed from the recipient mice and analyzed for both ovarian maturation and number and quality of existing oocytes.

### Nested PCR to detect GFP signal in oocytes

The presence of the GFP signal was determined using nested PCR, as previously described by others [48]. Briefly, for primary reactions, we used 4 µl of DNA template from a pool of isolated oocytes in a total volume of 15 µl. Cycling conditions consisted of an initial 3-min denaturation at 95°C, followed by 25 cycles, each consisting of a 30-sec denaturation at 94°C, a 45-sec annealing at 55°C, and a 1-min extension at 72°C. These 25 cycles were followed by a 7-min extension at 72°C. For the nested amplifications, we used 1.0 µl of the primary PCR product as the template in a total reaction volume of 12 µl. Nested cycling conditions were as described for the primary amplification, except that 35 cycles were used. Reaction products were subsequently analyzed by 2% agarose gel electrophoresis. Negative controls of oocytes from non-transgenic mice as well as a reverse transcriptase blank and a no-template blank were included in each experiment.

### Global gene expression analysis

For RNA isolation, PGCs were sorted by FACS, collected by centrifugation, lysed in RLT buffer (QIAGEN), and processed using the RNeasy micro and mini kits with on-column DNase digestion as per the manufacturer's instructions. Integrity of RNA samples was quality checked using a 2100 Bioanalyzer (Agilent). When possible, 300 ng of total RNA per sample were used as starting material for linear amplification (Illumina TotalPrep RNA Amplification Kit, Ambion), which involved synthesis of T7-linked double-stranded cDNA and 14 hr of *in vitro* transcription incorporating biotin-labeled nucleotides. Purified and labeled cRNA was then quality checked on a 2100 Bioanalyser and hybridized as biological or technical duplicates for 18 hr onto MouseRef-8 v2 gene expression BeadChips (Illumina), following the manufacturer's instructions. After being washed, the chips were stained with streptavidin-Cy3 (GE Healthcare) and scanned using an iScan reader and accompanying software.

### Microarray data processing

Bead intensities were mapped to gene information using BeadStudio 3.2 (Illumina). Background correction was performed using the Affymetrix Robust Multi-array Analysis (RMA) background correction model [49]. Variance stabilization was performed using log_2_ scaling, and gene expression normalization was calculated with the quantile method implemented in the lumi package of R-Bioconductor. Data post-processing and graphics were performed with in-house developed functions in Matlab. Hierarchical clustering of genes and samples was performed with a correlation metric and the Unweighted Pair-Group Method using Average (UPGMA) linkage method.

All the raw and processed microarray data discussed in this publication have been deposited into NCBI's Gene Expression Omnibus [50] and are accessible through GEO Series accession number GSE57506. (http://www.ncbi.nlm.nih.gov/geo/query/acc.cgi$acc=GSE57506).

## Supporting Information

Figure S1 **Global gene expression shows the** ***Gcnf*** **expression in different lines of mouse GSCs, compared with ESCs and germline-derived pluripotent stem cells (gPSCs).**
(EPS)

Figure S2 **Light microscopy of transplanted and non-transplanted GSCs into the testis.** (**A–B**) Tomato lentivirus–infected GSCs before transplantation into the seminiferous tubules of germ cell–depleted busulfan-treated mice. (**C–D**) GSCs without *Gcnf* siRNA (controls, only with tomato lentivirus); note the red GSCs have colonized the tubules. (**E–F**) GSCs with *Gcnf* siRNA; note the red GSCs have colonized the tubules. (**G–H**) Non-transplanted testicular tubules, showing no recovery of germ cells in germ cell–depleted testes 3 months after treatment with busulfan. Note that the scale bars are 20 uM.
(TIF)

Figure S3 **Expression of known germ cell markers and the newly identified genes in day-15** ***in vitro*** **–derived PGCs.** Real-time q-RT-PCR of pluripotency and known germ cell markers in (**A**) Oct4-GFP(ΔPE) ESC-derived PGCs and (**B**) *Gcnf*-deficient ESC-derived PGCs compared with ESCs. D denotes days. Real-time q-RT-PCR of the newly identified genes in (**C**) Oct4-GFP(ΔPE) ESC-derived PGCs and (**D**) *Gcnf*-deficient ESC-derived PGCs compared with ESCs.
(EPS)

Figure S4 **Efficiency of shRNA transfection in GSCs.** (**A–B**) FACS sorting plots, showing that more than 80% of the infected GSCs exhibited a positive tomato signal. (**C**) Depict the efficiency of shGcnf and showed the downregulation of gene in cells after 48 and 72 hours (75% downregulation of Gcnf after 72 hours).
(TIF)

Table S1 **Assay codes for TaqMan real-time q-RT-PCR.**
(TIF)

Table S2 **List of primers used for SYBR Green real-time q-RT-PCR.**
(TIF)

## References

[1] Chen F, Cooney AJ, Wang Y, Law SW, O'Malley BW (1994) Cloning of a novel orphan receptor (GCNF) expressed during germ cell development. Mol Endocrinol 8: 1434–1444. 7854358 10.1210/mend.8.10.7854358

[2] Fuhrmann G, Chung AC, Jackson KJ, Hummelke G, Baniahmad A, et al (2001) Mouse germline restriction of Oct4 expression by germ cell nuclear factor. Dev Cell 1: 377–387. 11702949 10.1016/s1534-5807(01)00038-7

[3] Lan ZJ, Chung AC, Xu X, DeMayo FJ, Cooney AJ (2002) The embryonic function of germ cell nuclear factor is dependent on the DNA binding domain. J Biol Chem 277: 50660–50667. 12381721 10.1074/jbc.M209586200

[4] Yan ZH, Medvedev A, Hirose T, Gotoh H, Jetten AM (1997) Characterization of the response element and DNA binding properties of the nuclear orphan receptor germ cell nuclear factor/retinoid receptor-related testis-associated receptor. J Biol Chem 272: 10565–10572. 9099702 10.1074/jbc.272.16.10565

[5] Schmitz TP, Susens U, Borgmeyer U (1999) DNA binding, protein interaction and differential expression of the human germ cell nuclear factor. Biochim Biophys Acta 1446: 173–180. 10524192 10.1016/s0167-4781(99)00079-2

[6] Gu P, LeMenuet D, Chung AC, Mancini M, Wheeler DA, et al (2005) Orphan nuclear receptor GCNF is required for the repression of pluripotency genes during retinoic acid-induced embryonic stem cell differentiation. Mol Cell Biol 25: 8507–8519. 16166633 10.1128/MCB.25.19.8507-8519.2005 PMC1265758

[7] Gu P, Xu X, Le Menuet D, Chung AC, Cooney AJ (2011) Differential recruitment of methyl CpG-binding domain factors and DNA methyltransferases by the orphan receptor germ cell nuclear factor initiates the repression and silencing of Oct4. Stem Cells 29: 1041–1051. 21608077 10.1002/stem.652 PMC3468724

[8] Chung AC, Katz D, Pereira FA, Jackson KJ, DeMayo FJ, et al (2001) Loss of orphan receptor germ cell nuclear factor function results in ectopic development of the tail bud and a novel posterior truncation. Mol Cell Biol 21: 663–677. 11134352 10.1128/MCB.21.2.663-677.2001 PMC86646

[9] Susens U, Aguiluz JB, Evans RM, Borgmeyer U (1997) The germ cell nuclear factor mGCNF is expressed in the developing nervous system. Dev Neurosci 19: 410–420. 9323461 10.1159/000111238

[10] Katz D, Niederberger C, Slaughter GR, Cooney AJ (1997) Characterization of germ cell-specific expression of the orphan nuclear receptor, germ cell nuclear factor. Endocrinology 138: 4364–4372. 9322952 10.1210/endo.138.10.5444

[11] Lan ZJ, Gu P, Xu X, Cooney AJ (2003) Expression of the orphan nuclear receptor, germ cell nuclear factor, in mouse gonads and preimplantation embryos. Biol Reprod 68: 282–289. 12493724 10.1095/biolreprod.102.008151

[12] Agoulnik IY, Cho Y, Niederberger C, Kieback DG, Cooney AJ (1998) Cloning, expression analysis and chromosomal localization of the human nuclear receptor gene GCNF. FEBS Lett 424: 73–78. 9537518 10.1016/s0014-5793(98)00142-2

[13] Hummelke GC, Cooney AJ (2001) Germ cell nuclear factor is a transcriptional repressor essential for embryonic development. Front Biosci 6: D1186–1191. 11578963 10.2741/hummelke

[14] Hummelke GC, Meistrich ML, Cooney AJ (1998) Mouse protamine genes are candidate targets for the novel orphan nuclear receptor, germ cell nuclear factor. Mol Reprod Dev 50: 396–405. 9669523 10.1002/(SICI)1098-2795(199808)50:4<396::AID-MRD3>3.0.CO;2-D

[15] Braat AK, Zandbergen MA, De Vries E, Van Der Burg B, Bogerd J, et al (1999) Cloning and expression of the zebrafish germ cell nuclear factor. Mol Reprod Dev 53: 369–375. 10398411 10.1002/(SICI)1098-2795(199908)53:4<369::AID-MRD1>3.0.CO;2-U

[16] Joos TO, David R, Dreyer C (1996) xGCNF, a nuclear orphan receptor is expressed during neurulation in Xenopus laevis. Mech Dev 60: 45–57. 9025060 10.1016/s0925-4773(96)00599-0

[17] Lan ZJ, Gu P, Xu X, Jackson KJ, DeMayo FJ, et al (2003) GCNF-dependent repression of BMP-15 and GDF-9 mediates gamete regulation of female fertility. EMBO J 22: 4070–4081. 12912906 10.1093/emboj/cdg405 PMC175795

[18] Chung AC, Cooney AJ (2001) Germ cell nuclear factor. Int J Biochem Cell Biol 33: 1141–1146. 11606249 10.1016/s1357-2725(01)00081-4

[19] Chung AC, Xu X, Niederreither KA, Cooney AJ (2006) Loss of orphan nuclear receptor GCNF function disrupts forebrain development and the establishment of the isthmic organizer. Dev Biol 293: 13–24. 16530751 10.1016/j.ydbio.2005.12.017

[20] Akamatsu W, DeVeale B, Okano H, Cooney AJ, van der Kooy D (2009) Suppression of Oct4 by germ cell nuclear factor restricts pluripotency and promotes neural stem cell development in the early neural lineage. J Neurosci 29: 2113–2124. 19228964 10.1523/JNEUROSCI.4527-08.2009 PMC6666351

[21] Kehler J, Tolkunova E, Koschorz B, Pesce M, Gentile L, et al (2004) Oct4 is required for primordial germ cell survival. EMBO Rep 5: 1078–1083. 15486564 10.1038/sj.embor.7400279 PMC1299174

[22] Yeom YI, Fuhrmann G, Ovitt CE, Brehm A, Ohbo K, et al (1996) Germline regulatory element of Oct-4 specific for the totipotent cycle of embryonal cells. Development 122: 881–894. 8631266 10.1242/dev.122.3.881

[23] Yoshimizu T, Sugiyama N, De Felice M, Yeom YI, Ohbo K, et al (1999) Germline-specific expression of the Oct-4/green fluorescent protein (GFP) transgene in mice. Dev Growth Differ 41: 675–684. 10646797 10.1046/j.1440-169x.1999.00474.x

[24] Ko K, Tapia N, Wu G, Kim JB, Bravo MJ, et al (2009) Induction of pluripotency in adult unipotent germline stem cells. Cell Stem Cell 5: 87–96. 19570517 10.1016/j.stem.2009.05.025

[25] Ogawa T, Dobrinski I, Avarbock MR, Brinster RL (2000) Transplantation of male germ line stem cells restores fertility in infertile mice. Nat Med 6: 29–34. 10613820 10.1038/71496 PMC4879876

[26] Sabour D, Arauzo-Bravo MJ, Hubner K, Ko K, Greber B, et al (2011) Identification of genes specific to mouse primordial germ cells through dynamic global gene expression. Hum Mol Genet 20: 115–125. 20940145 10.1093/hmg/ddq450

[27] Ventura A, Kirsch DG, McLaughlin ME, Tuveson DA, Grimm J, et al (2007) Restoration of p53 function leads to tumour regression in vivo. Nature 445: 661–665. 17251932 10.1038/nature05541

[28] Hochedlinger K, Yamada Y, Beard C, Jaenisch R (2005) Ectopic expression of Oct-4 blocks progenitor-cell differentiation and causes dysplasia in epithelial tissues. Cell 121: 465–477. 15882627 10.1016/j.cell.2005.02.018

[29] Barreto G, Borgmeyer U, Dreyer C (2003) The germ cell nuclear factor is required for retinoic acid signaling during Xenopus development. Mech Dev 120: 415–428. 12676320 10.1016/s0925-4773(03)00018-2

[30] Hirose T, O'Brien DA, Jetten AM (1995) RTR: a new member of the nuclear receptor superfamily that is highly expressed in murine testis. Gene 152: 247–251. 7835709 10.1016/0378-1119(94)00656-d

[31] Yang G, Zhang YL, Buchold GM, Jetten AM, O'Brien DA (2003) Analysis of germ cell nuclear factor transcripts and protein expression during spermatogenesis. Biol Reprod 68: 1620–1630. 12606326 10.1095/biolreprod.102.012013

[32] Zhang YL, Akmal KM, Tsuruta JK, Shang Q, Hirose T, et al (1998) Expression of germ cell nuclear factor (GCNF/RTR) during spermatogenesis. Mol Reprod Dev 50: 93–102. 9547515 10.1002/(SICI)1098-2795(199805)50:1<93::AID-MRD12>3.0.CO;2-Z

[33] Hummelke GC, Cooney AJ (2004) Reciprocal regulation of the mouse protamine genes by the orphan nuclear receptor germ cell nuclear factor and CREMtau. Mol Reprod Dev 68: 394–407. 15236322 10.1002/mrd.20092

[34] Anderson R, Copeland TK, Scholer H, Heasman J, Wylie C (2000) The onset of germ cell migration in the mouse embryo. Mech Dev 91: 61–68. 10704831 10.1016/s0925-4773(99)00271-3

[35] Ginsburg M, Snow MH, McLaren A (1990) Primordial germ cells in the mouse embryo during gastrulation. Development 110: 521–528. 2133553 10.1242/dev.110.2.521

[36] Lawson KA, Hage WJ (1994) Clonal analysis of the origin of primordial germ cells in the mouse. Ciba Found Symp 182: 68–84 discussion 84–91. 7835158 10.1002/9780470514573.ch5

[37] Molyneaux KA, Stallock J, Schaible K, Wylie C (2001) Time-lapse analysis of living mouse germ cell migration. Dev Biol 240: 488–498. 11784078 10.1006/dbio.2001.0436

[38] Saitou M, Barton SC, Surani MA (2002) A molecular programme for the specification of germ cell fate in mice. Nature 418: 293–300. 12124616 10.1038/nature00927

[39] Okumura LM, Lesch BJ, Page DC (2013) The ligand binding domain of GCNF is not required for repression of pluripotency genes in mouse fetal ovarian germ cells. PLoS One 8: e66062. 23762465 10.1371/journal.pone.0066062 PMC3676325

[40] Koubova J, Menke DB, Zhou Q, Capel B, Griswold MD, et al (2006) Retinoic acid regulates sex-specific timing of meiotic initiation in mice. Proc Natl Acad Sci U S A 103: 2474–2479. 16461896 10.1073/pnas.0510813103 PMC1413806

[41] Oulad-Abdelghani M, Bouillet P, Decimo D, Gansmuller A, Heyberger S, et al (1996) Characterization of a premeiotic germ cell-specific cytoplasmic protein encoded by Stra8, a novel retinoic acid-responsive gene. J Cell Biol 135: 469–477. 8896602 10.1083/jcb.135.2.469 PMC2121034

[42] Anderson EL, Baltus AE, Roepers-Gajadien HL, Hassold TJ, de Rooij DG, et al (2008) Stra8 and its inducer, retinoic acid, regulate meiotic initiation in both spermatogenesis and oogenesis in mice. Proc Natl Acad Sci U S A 105: 14976–14980. 18799751 10.1073/pnas.0807297105 PMC2542382

[43] Baltus AE, Menke DB, Hu YC, Goodheart ML, Carpenter AE, et al (2006) In germ cells of mouse embryonic ovaries, the decision to enter meiosis precedes premeiotic DNA replication. Nat Genet 38: 1430–1434. 17115059 10.1038/ng1919

[44] Mark M, Jacobs H, Oulad-Abdelghani M, Dennefeld C, Feret B, et al (2008) STRA8-deficient spermatocytes initiate, but fail to complete, meiosis and undergo premature chromosome condensation. J Cell Sci 121: 3233–3242. 18799790 10.1242/jcs.035071

[45] Ogawa T, Arechaga JM, Avarbock MR, Brinster RL (1997) Transplantation of testis germinal cells into mouse seminiferous tubules. Int J Dev Biol 41: 111–122. 9074943

[46] Hubner K, Fuhrmann G, Christenson LK, Kehler J, Reinbold R, et al (2003) Derivation of oocytes from mouse embryonic stem cells. Science 300: 1251–1256. 12730498 10.1126/science.1083452

[47] Peters AH, Plug AW, van Vugt MJ, de Boer P (1997) A drying-down technique for the spreading of mammalian meiocytes from the male and female germline. Chromosome Res 5: 66–68. 9088645 10.1023/a:1018445520117

[48] Wu G, Hao L, Han Z, Gao S, Latham KE, et al (2005) Maternal transmission ratio distortion at the mouse Om locus results from meiotic drive at the second meiotic division. Genetics 170: 327–334. 15744049 10.1534/genetics.104.039479 PMC1449735

[49] Irizarry RA, Bolstad BM, Collin F, Cope LM, Hobbs B, et al (2003) Summaries of Affymetrix GeneChip probe level data. Nucleic Acids Research 31. 10.1093/nar/gng015 PMC150247 12582260

[50] Edgar R, Domrachev M, Lash AE (2002) Gene Expression Omnibus: NCBI gene expression and hybridization array data repository. Nucleic Acids Research 30: 207–210. 11752295 10.1093/nar/30.1.207 PMC99122

