# Supplementary figures and images for: Germ Cell Nuclear Factor Regulates Gametogenesis in Developing Gonads
Source: PLoS One. 2014 Aug 20;9(8):e103985. doi: 10.1371/journal.pone.0103985 (PMC4139263; doi:10.1371/journal.pone.0103985)

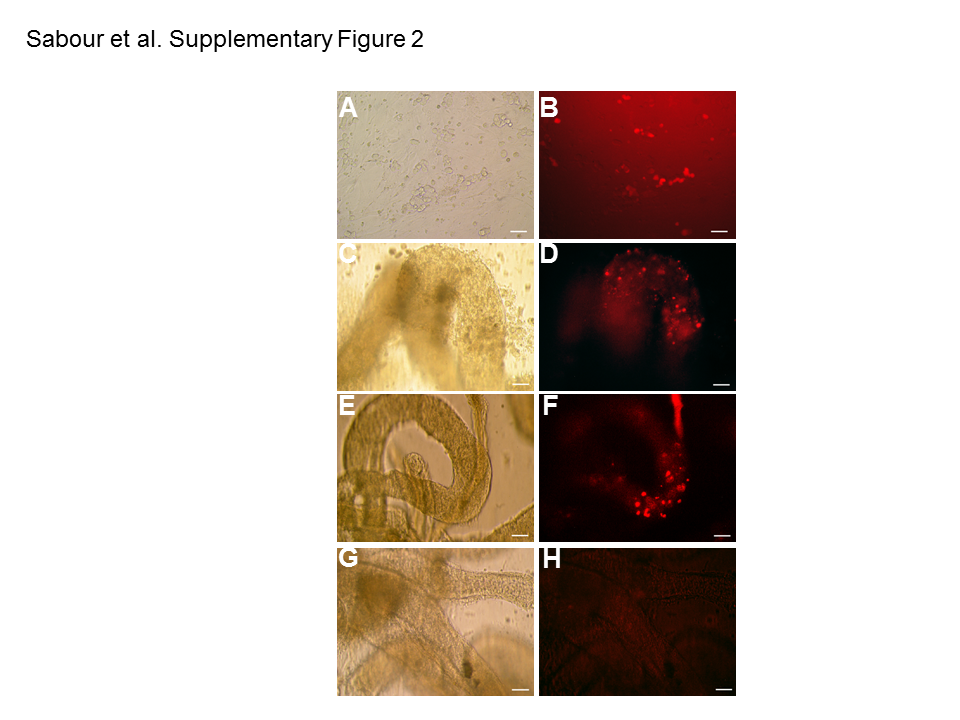

Supplement: Figure S2 — Light microscopy of transplanted and non-transplanted GSCs into the testis. (A–B) Tomato lentivirus–infected GSCs before transplantation into the seminiferous tubules of germ cell–depleted busulfan-treated mice. (C–D) GSCs without Gcnf siRNA (controls, only with tomato lentivirus); note the red GSCs have colonized the tubules. (E–F) GSCs with Gcnf siRNA; note the red GSCs have colonized the tubules. (G–H) Non-transplanted testicular tubules, showing no recovery of germ cells in germ cell–depleted testes 3 months after treatment with busulfan. Note that the scale bars are 20 uM. (TIF) [file pone.0103985.s002.tif]

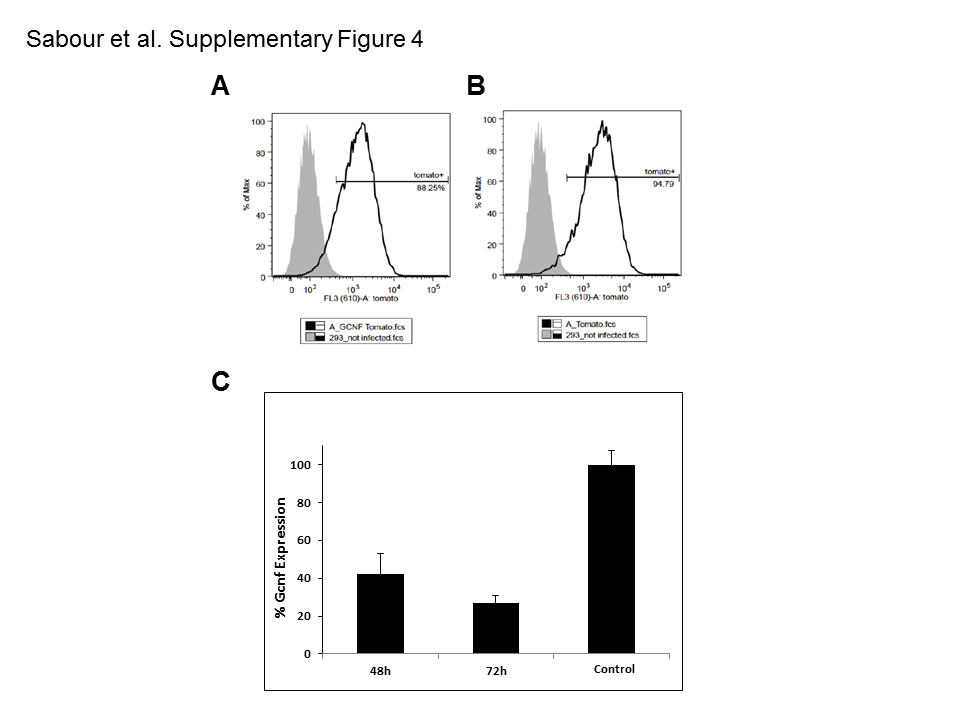

Supplement: Figure S4 — Efficiency of shRNA transfection in GSCs. (A–B) FACS sorting plots, showing that more than 80% of the infected GSCs exhibited a positive tomato signal. (C) Depict the efficiency of shGcnf and showed the downregulation of gene in cells after 48 and 72 hours (75% downregulation of Gcnf after 72 hours). (TIF) [file pone.0103985.s004.tif]

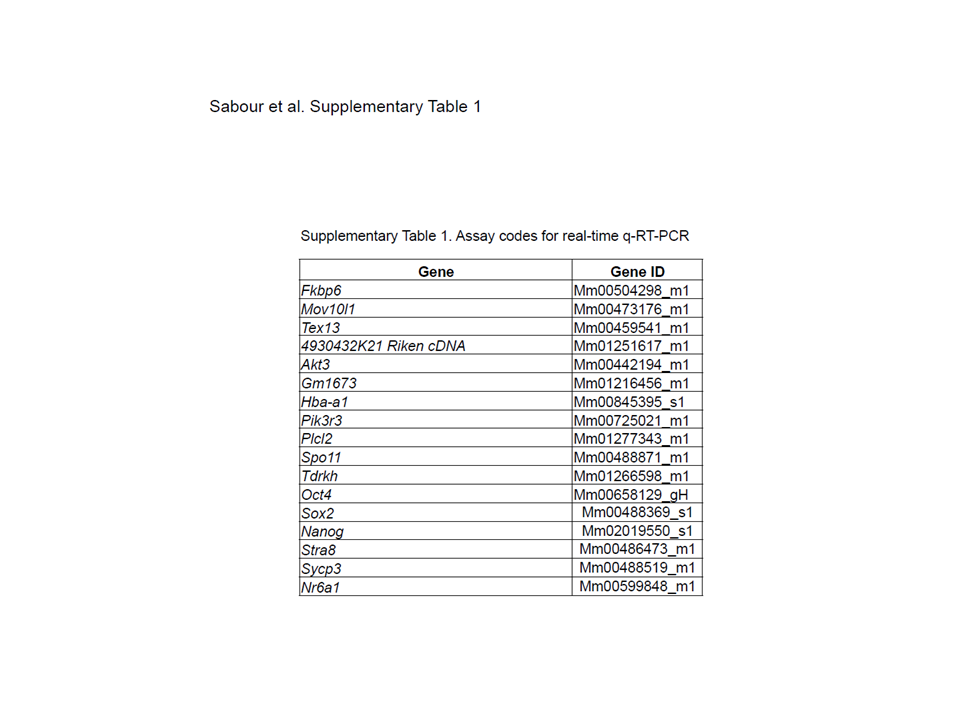

Supplement: Table S1 — Assay codes for TaqMan real-time q-RT-PCR. (TIF) [file pone.0103985.s005.tif]

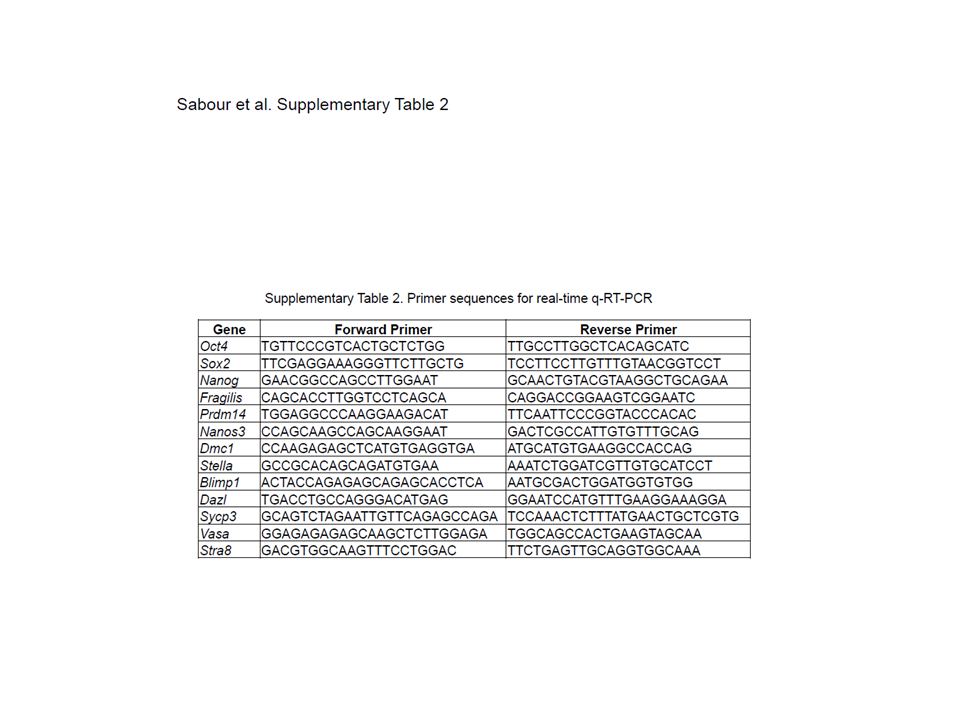

Supplement: Table S2 — List of primers used for SYBR Green real-time q-RT-PCR. (TIF) [file pone.0103985.s006.tif]
